# Explicit Nonlinear Finite Element Geometric Analysis of Parabolic Leaf Springs under Various Loads

**DOI:** 10.1155/2013/261926

**Published:** 2013-11-03

**Authors:** Y. S. Kong, M. Z. Omar, L. B. Chua, S. Abdullah

**Affiliations:** ^1^Department of Mechanical & Materials Engineering, Faculty of Engineering & Built Environment, Universiti Kebangsaan Malaysia (UKM), 43600 Bangi, Selangor, Malaysia; ^2^APM Engineering & Research Sdn Bhd, Level 4, Bangunan B, Peremba Square, Saujana Resort, Seksyen U2, 40150 Shah Alam, Selangor, Malaysia; ^3^Center for Automotive Research, Faculty of Engineering & Built Environment, Universiti Kebangsaan Malaysia (UKM), 43600 Bangi, Selangor, Malaysia

## Abstract

This study describes the effects of bounce, brake, and roll behavior of a bus toward its leaf spring suspension systems. Parabolic leaf springs are designed based on vertical deflection and stress; however, loads are practically derived from various modes especially under harsh road drives or emergency braking. Parabolic leaf springs must sustain these loads without failing to ensure bus and passenger safety. In this study, the explicit nonlinear dynamic finite element (FE) method is implemented because of the complexity of experimental testing A series of load cases; namely, vertical push, wind-up, and suspension roll are introduced for the simulations. The vertical stiffness of the parabolic leaf springs is related to the vehicle load-carrying capability, whereas the wind-up stiffness is associated with vehicle braking. The roll stiffness of the parabolic leaf springs is correlated with the vehicle roll stability. To obtain a better bus performance, two new parabolic leaf spring designs are proposed and simulated. The stress level during the loadings is observed and compared with its design limit. Results indicate that the newly designed high vertical stiffness parabolic spring provides the bus a greater roll stability and a lower stress value compared with the original design. Bus safety and stability is promoted, as well as the load carrying capability.

## 1. Introduction

Leaf springs are widely used in the automotive industry as primary components in suspension systems for heavy vehicles because they possess advantages such as a simple structure, excellent guiding effects, convenience in maintenance, low cost, and prone to axle location. Leaf spring designs are mainly based on simplified equations as well as trial-and-error methods. Simplified equation models are limited to three-link mechanism assumptions and linear beam theory. According to beam deflection theory, the deflection of a beam is based on the dimensional, cross-sectional profile of the current beam. The thickness of the cross-sectional profile of a parabolic leaf spring contributes to the stiffness in the vertical direction. The higher vertical stiffness of the leaf spring provides vehicles additional load-carrying capabilities. Leaf springs could be categorized into two types: multileaf and parabolic leaf. From a geometric perspective, a parabolic leaf spring has a constant width but decreasing thickness from the center of its line of encasement in a parabolic profile, whereas a multileaf spring maintains a constant thickness along its length [1]. Parabolic springs are predicted to perform more efficiently compared with traditional multi-leaf springs because the former is lightweight and has less friction between steel leaves. 

Leaf springs absorb and store energy and then release it. The characteristics of a spring suspension are chiefly influenced by the spring vertical stiffness and the static deflection of the spring. The ride frequency and the load-carrying capabilities of the leaf spring vehicle are affected by the vertical stiffness of the installed leaf springs. The vertical stiffness of a leaf spring is defined as the change in load per unit deflection in the vertical direction. Most leaf springs are designed to operate with respect to the vertical loading of the vehicle. However, leaf springs are practically loaded not only by vertical forces but also by horizontal forces and torques in the longitudinal directions. The center of a spring is elastically constrained against wind-up or rotation torque along a longitudinal vertical plane because of its wind-up stiffness. Leaf spring wind-up usually occurs while the vehicle brakes and accelerates. When a car suddenly starts or stops, front-down or rear-down postures impose a rotational torque on the spring, referred to as a wind-up torque [1]. In addition, leaf springs also sustain torsional load where the moment generated from the vertical lateral plane when the vehicle rolls. 

Several studies have been conducted on leaf spring analysis such as deflection and stress analysis by using the finite element method (FEM) [2–6]. The vertical stiffness and stress analysis conducted is based on the vertical loading of the leaf spring. Kong et al. performed a simulation of leaf springs on the basis of vertical and longitudinal loading [7]. Qin et al. published a research article on multi-leaf spring and Hotchkiss suspension analysis [8]. Leaf spring under varying load cases such as vertical push, wind-up, roll, and cornering analysis was demonstrated in the analysis. The simulation results provided the vertical, wind-up, and roll stiffness of the leaf spring suspension system. Savaidis et al. evaluated the severe braking conditions of the axle [9]. The mechanical stress-strain behavior of the leaf springs was calculated by FEM analysis. Another elastic leaf spring model was also developed for multi-body vehicle systems of a sport utility vehicle to simulate the axle wind-up under severe braking [10]. A nonlinear FE formulation based on the floating frame of reference approach was introduced with a full FE model of leaf springs with contact and friction. When contact and friction are considered, nonlinear model analysis is considered instead of linear analysis. For nonlinear model analysis, various models such as gun control system were optimized through Pareto optimal solution [11] and electrohydrostatic actuator through signal compression method [12]. The nonlinear model is preferred to be solved in dynamic scheme where static analysis could not encounter the friction, material, and geometric nonlinearities. 

The most implemented algorithms in dynamic FE analysis (FEA) are the implicit and the explicit schemes. In implicit dynamic simulation, an extension of the Newmark method known as *α*-HHT is used as a default time integrator [13]. Mousseau et al. implemented the implicit dynamic schemes to predict the handling performance of a vehicle [14]. This approach is time efficient and yields reasonable results. However, the explicit dynamic method derived from the Newmark scheme was also widely adopted in dynamic analysis [15, 16]. An explicit dynamic simulation for the stamping part of automotive components was performed [17]. The explicit method shows stability of convergence during simulation. Both the implicit and the explicit methods have their pros and cons. The explicit technique entails a lower cost; however, given a slow case, the solutions are unstable. Given the same condition, the implicit method provides more accurate results [18]. The simulation of the crimping process, which uses both the implicit and the explicit techniques, was conducted by Kugener [19]. The simulation results indicated that the explicit method is superior to the implicit method especially when numerous contacts are considered. Other than the mentioned two schemes, it is worth mentioning that the new developed approach semi-implicit finite difference scheme is implemented to analyze the second law of thermodynamics of fluid [20].

The design of a parabolic leaf spring in a bus presents a challenge to engineers given very complex and limited considerations. Road conditions and the driving behavior of the drivers subject the leaf springs to varying loading conditions, at times severely damaging the leaf springs. Currently, leaf spring designs focus solely on the load-carrying capabilities or relative vertical stiffness. As mentioned in other previous studies, the design of the leaf spring with vertical stiffness only is insufficient when catastrophic failures have the possibility of occurrence. However, the experimental methods verifying the stress under those varying loading modes are too costly and complex to perform. This paper aims to present the analysis of the stress level of the parabolic leaf springs under different loading conditions by computer-aided engineering. The failure modes of the leaf springs normally occur under harsh braking or suspension rolling while striking a pothole. The braking condition of the bus is associated with the leaf spring wind-up, whereas pothole striking is related to the suspension roll. To promote bus safety under such conditions, newly designed parabolic leaf springs are evaluated in simulations for their performance. The new leaf spring designs are expected to provide enhanced roll resistance, improved load-carrying capability, and reduced occurrence of potential failure.

## 2. FE Explicit Model

**Figure 1:**
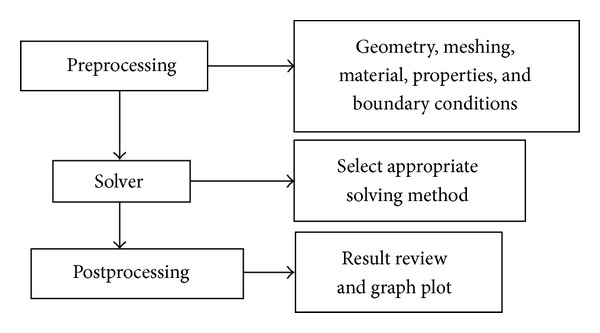
Typical FEA procedures by commercial software.

The standard simulation setup for any commercial FEA software is shown in Figure 1. As seen in Figure 1, the simulation can be divided into three categories: preprocessing, solving, and postprocessing. First, computer-aided design models are generated for FE meshing. In this study, a manual hexahedra element mesh is applied for the stress analysis of the parabolic springs. To obtain good simulation results, the quality of the mesh is optimized by the element quality index. The materials and properties of the leaf springs and silencers have also been assigned, and these details are shown in Table 1. Boundary conditions to simulate the degree of freedom of the leaf springs under varying loading conditions differ. 

FE procedures need to be well developed to perform a complex FE nonlinear analysis. Selection of the appropriate solving method is significant. A conditionally stable explicit integration scheme derived from the Newmark scheme from the RADIOSS solver has been introduced (RADIOSS is a copyright of Altair Hyperworks, Altair Engineering Inc.). In dynamic analysis, the equation of motion for discrete structural models is expressed as follows:

(1) $$\begin{array}{c} M\ddot{u}+C\dot{u}+ku=F, \end{array}$$

where *M*, *C*, and *K* represent the mass, viscous damping, and stiffness matrices. $\ddot{u}$, $\dot{u}$, and *u* denote the displacement, velocity, and acceleration vectors, respectively. *F* is the external force vector. In the general Newmark method, the state vector is computed as follows:

(2) $$\begin{array}{c} u_{t+1}=u_{t}+{\Delta tu}_{t}+{\dot{u}}_{t}+\left(\frac{1}{2}-\beta \right){\Delta t}^{2}{\ddot{u}}_{t}+\beta \Delta t^{2}{\ddot{u}}_{t+1},\\ {\dot{u}}_{t+1}={\dot{u}}_{t}+\Delta t\left[\left(1-\gamma \right)\ddot{u}+{\gamma \;\ddot{u}}_{t+1}\right], \end{array}$$

where *β* and *γ* are the specified coefficients that govern the stability, accuracy, and numerical dissipation of the integration method [16]. A conditionally stable explicit integration scheme can be derived from the Newmark scheme given the following:

(3) $$\begin{array}{c} {\dot{u}}_{t+1}={\dot{u}}_{t}+\frac{1}{2}{\Delta t}^{2}\left({\ddot{u}}_{t}+{\ddot{u}}_{t+1}\right),\\ u_{t+1}=u_{t}+{\Delta tu}_{t}+\frac{1}{2}{\Delta t}^{2}{\ddot{u}}_{t}. \end{array}$$

The explicit central difference integration scheme can be derived from the relationships. The central difference scheme is used when explicit analysis is selected. The time step must be smaller than the critical time step to ensure the stability of the solution. Newmark nonlinear analysis efficiently captures energy decay and exhibits a satisfactory long-term performance after being tested [15].

To reduce the dynamic effects, dynamic relaxation is used in the explicit scheme. A diagonal damping matrix proportional to the mass matrix is added to the dynamic equation

(4) $$\begin{array}{c} \left[C\right]=\frac{2\beta }{T}\left[M\right], \end{array}$$

where *β* is the relaxation value and *T* is the period to be damped. Thus, a viscous stress tensor is added to the stress tensor. In an explicit code, the application of the dashpot force modifies the velocity equation without relaxation

(5) $$\begin{array}{c} V_{t+\Delta t/2}=V_{t-\Delta t/2}+\gamma_{t}\Delta_{t} \end{array}$$

to velocity equation with relaxation

(6) $$\begin{array}{c} V_{t+\Delta t/2}=\left(1-2\omega \right)V_{t-\Delta t/2}+\left(1-\varpi \right)\gamma_{t}\Delta_{t}, \end{array}$$

where

(7) $$\begin{array}{c} \omega =\beta \frac{\Delta t}{t}. \end{array}$$

When this option is activated, the running time of the whole simulation is increased. However, the damping period for the system is controlled within acceptable limits.

## 3. Contacts and Load Cases

**Figure 2:**
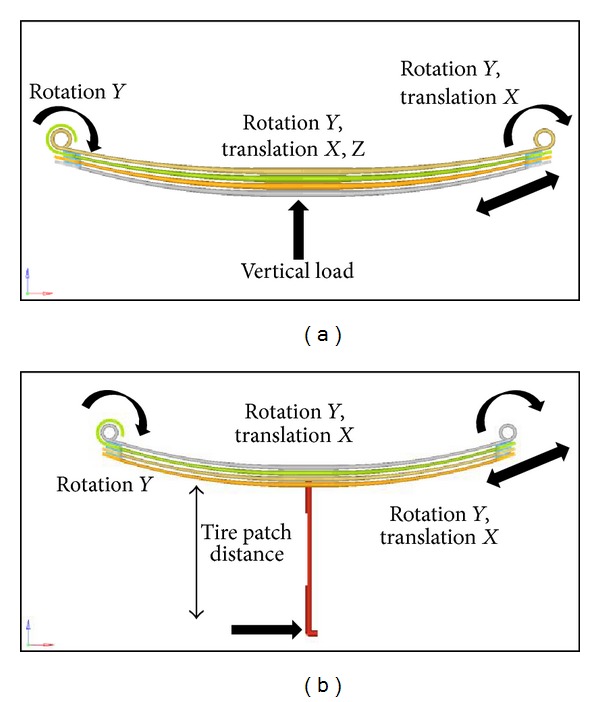
Boundary conditions and loads applied: (a) vertical push, (b) wind-up.

Three different parabolic leaf spring designs were analyzed in this study. Each design was simulated with different loading cases. Therefore, different simulation boundary condition setups for the vertical push, wind-up, and roll suitable to the load case were conducted accordingly. First, the boundary conditions for the vertical push were performed with free rotation around the *y*-axis for the front eye, whereas the rear eye was constrained in the *Y*, *Z* translation and the *X*, *Z* rotation. The boundary conditions are complied with [21]. The center of the spring was allowed only in the *X*-*Z* translation and the *Y* rotation. The vertical push boundary condition setup is shown in Figure 2(a). For the wind-up load case setup, the applied boundary conditions for the eye were similar to the vertical push with free rotation around the *y* axis for the front eye, whereas the rear eye was constrained in the *Y*-*Z* translation and the *X*-*Z* rotation. After maximum vertical loading is applied, a longitudinal force was created and applied at the center of the parabolic leaf springs [22]. The wind-up establishment of the parabolic leaf springs is illustrated in Figure 2(b).

**Figure 3:**
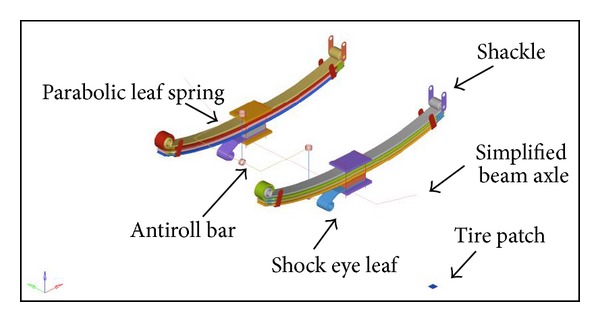
Roll load case simulation model.

For the suspension roll study, loads are applied to push the suspension to a curb position. A moment is subsequently applied to the suspension by increasing the vertical load on the left side and decreasing the load on the right side [8]. The leaf spring is expected to hit the jounce stopper after a 40 mm displacement is imposed. In this case, the load is applied at the tire patch that represents the contacts of tire to the ground. The boundary condition of the parabolic leaf spring can freely rotate around the *Y* axis for the front eye, whereas the rear eye is attached to the shackle, and the shackle can rotate in the *y*-axis only. The front module of conventional buses considered in this study employed an antiroll bar to enhance the roll stiffness of the vehicle. The antiroll bar can be idealized as a torsional stiffener connected between the sprung and the unsprung masses. When the roll bar undergoes a relative rotation between the two masses, a restoring moment, *M*_Φ_, is generated, which is then related to its roll stiffness *k*_Φ_ [23]. The part of the antiroll bar that is connected to the vehicle sprung mass is fixed in all degrees of freedom. The total setup of the suspension roll model is shown in Figure 3.

Some parabolic leaf springs are designed to endure vertical load, whereas others are also designed to sustain wind-up loads. The vertical rate of the spring is calculated based on the beam deflection theory. The formula for the vertical rate *k* for parabolic leaf springs is indicated [1] as follows:

(8) $$\begin{array}{c} K=\frac{Ew_{o}t_{o}^{3}}{4l^{3}}\times C_{v}, \end{array}$$

where *E* is the spring material elastic modulus, *t*_*o*_ is the thickness at center of the spring, *w*_*o*_ is the width at the center of the spring, *l* is the length of cantilever, and *C*_*v*_ is the vertical rate factor. Besides that, lateral rate of the parabolic leaf spring is also taken into design considerations. The wind-up stiffness, *ω* is predicted through the vertical stiffness of the leaf spring as shown in equation [1] as follows:

(9) $$\begin{array}{c} \omega =\frac{kl_{2}}{4}. \end{array}$$

In geometric nonlinear analysis, components will undergo large deformations. The nonlinearities always come from contact or materials. A general purpose contact is introduced in Radioss which is FE commercial software. The interface stiffness, *I*_*s*_, is computed from both the masters, *K*_*m*_, and slaves segment, *K*_*s*_. The interface stiffness relationship between the master and slave is defined in equation

(10) $$\begin{array}{c} I_{s}=\frac{k_{m}\times k_{s}}{\left(k_{m}+k_{s}\right)}. \end{array}$$

Friction formulation is also being introduced in this contact interface. The most well-known friction law is the Coulomb friction law. This formulation provides accurate results with just one input parameter which is Coulomb friction coefficient, *μ* [24].

## 4. Result and Discussions

**Figure 4:**
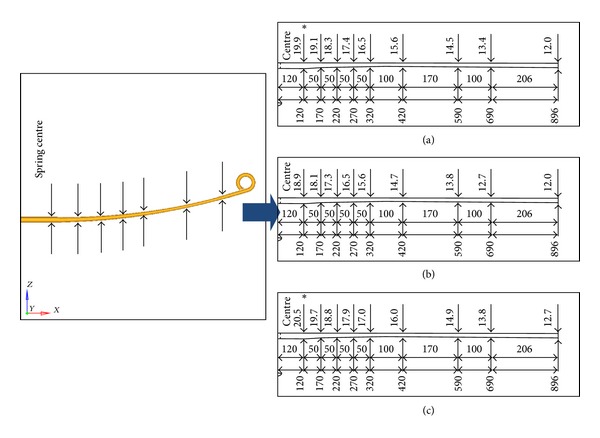
Taper profile of (a) Baseline, (b) Iteration 1, and (c) Iteration 2.

**Figure 5:**
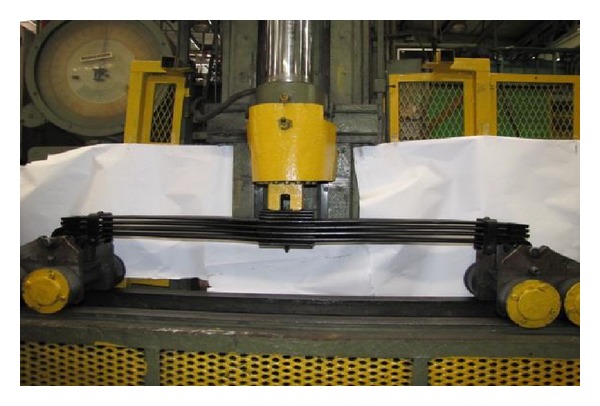
Experimental vertical stiffness test for leaf springs.

**Figure 6:**
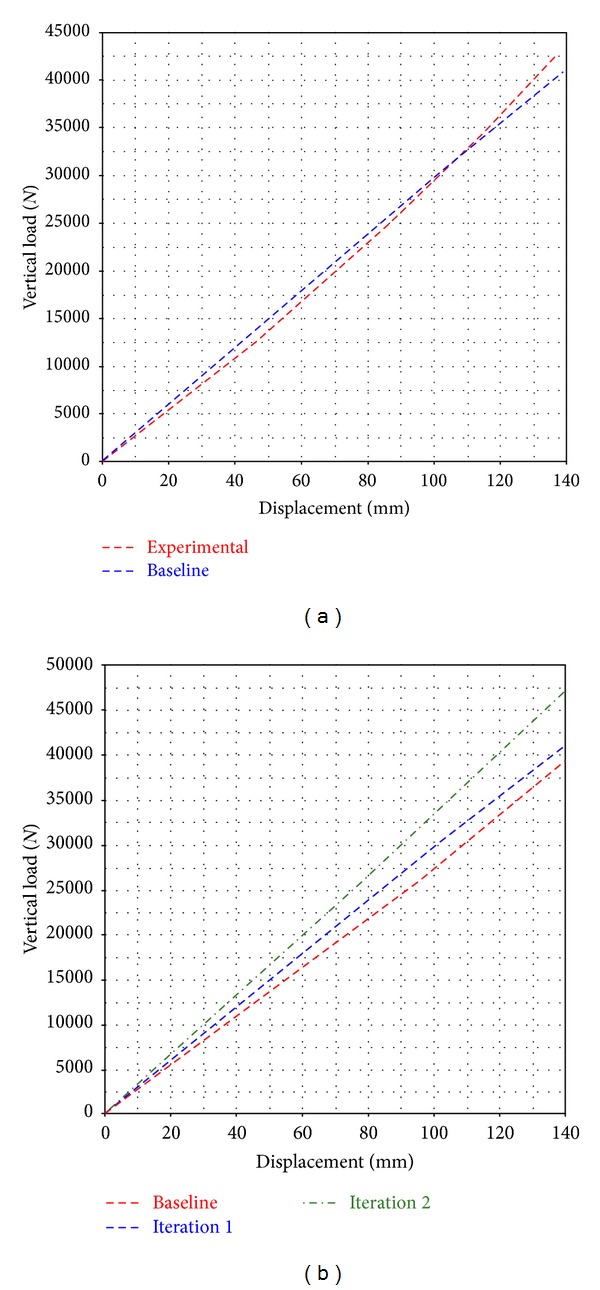
Graph of vertical stiffness comparison: (a) Baseline and experimental, (b) Baseline, Iteration 1, and Iteration 2.

Three parabolic leaf spring designs were prepared and simulated for validation purpose. One of the front parabolic leaf springs was obtained from the original bus model as benchmark for the analysis. The original parabolic leaf spring was named as “Baseline” in the simulation case. The profile design of “Baseline” is shown in Figure 4(a). The new parabolic leaf spring designs are named as “Iteration 1” and “Iteration 2,” respectively where the designs are shown in Figures 4(b) and 4(c), respectively. To obtain a proper spring characteristic of the Baseline model parabolic leaf spring, an experimental testing has been conducted. The experimental setup is shown in Figure 5 [25]. A vertical load is applied from the centre of the leaf spring while the displacement at the centre is measured. The front and rear eye of the parabolic spring are allowed to rotate in in lateral axis and translate in longitudinal axis. The gradient of the force versus deflection curve is the vertical stiffness of the spring. The simulation result of Baseline model is compared to the experimental result for correlation purpose as shown in Figure 6(a). From Figure 6(a), the vertical stiffness of the tested experimental parabolic leaf spring is 311 N/mm while the simulation model is 295 N/mm. It can be concluded that the simulation model and experimental test have a 95% good correlation. After that, vertical stiffness of Iterations 1 and 2 parabolic leaf springs is also plotted and compared to baseline model as shown in Figure 6(b). As seen in Figure 6(b), parabolic leaf spring of Iteration 1 has vertical stiffness of 281 N/mm while the parabolic leaf spring of Iteration 2 is 338 N/mm. The vertical stiffness of the leaf springs plays important role in determining the vehicle load-carrying capability. As the vertical stiffness of the leaf spring is higher, the load capacity of the vehicle will also be greater. In order to examine the load capabilities and stability of designed parabolic leaf springs toward original design, the parabolic leaf springs in Iterations 1 and 2 should have different vertical stiffnesses. The parabolic leaf spring in Iteration 1 has lower vertical stiffness which means lower load-carrying capability while the Iteration 2 has the greater vertical stiffness compared to the original parabolic leaf spring design (Baseline).

**Figure 7:**
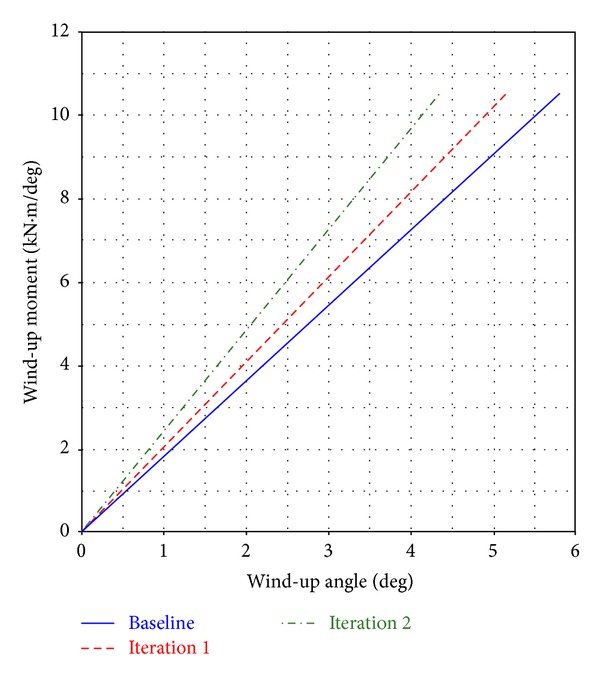
Comparison of wind-up moment versus wind-up angle curve.

When a car suddenly starts or stops, front-down or rear-down posture occurs, imposing a rotational torque or “wind-up torque” on the leaf spring [22]. Leaf springs experience longitudinal loading, in addition to vertical stiffness, especially when the vehicle brakes or accelerates. Meanwhile, wind-up analysis is performed in two stages. In the first stage, the spring is pushed to a vertical curb position; in the second stage, a longitudinal load is applied on the leaf spring center. The situation is considerably more difficult in case of braking. The acting brake force yields an “S-” shaped deformation of the leaf spring. This “S” deformation changes the kinematics of the front axle system, resulting in unwelcome swerving of the vehicle [9]. Such deformation is particularly undesirable because the moment of the inertia of the axle around the *y* axis can lead to periodic deformations, where the axle accepts a torque higher than the friction limit for a short time and then slips when the inertial force disappears. Vibration and loss of braking efficiency or traction then occur [26]. Therefore, the deformation of the “S” shape during braking is undesirable. To predict the wind-up stiffness of the parabolic leaf spring, aft load is applied to the tire patch to obtain the wind-up moment versus the angle curve, as shown in Figure 7. In Figure 7, the wind-up stiffness of the parabolic leaf spring in the Baseline is 1.82 kN·m/degree, whereas that in Iteration 1 is 2.04 kN·m/degree. The wind-up stiffness of the parabolic leaf spring in Iteration 2 is 2.42 kN·m/degree, indicating that Iteration 2 has a higher wind-up stiffness compared with Iteration 1 and the Baseline. This result suggests that “S” deformation is reduced under the same braking condition.

**Figure 8:**
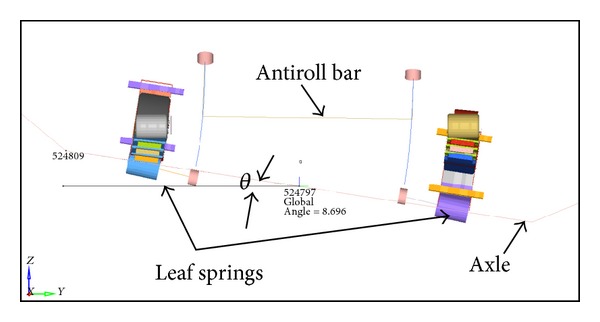
Roll angle measurement of suspension system.

**Figure 9:**
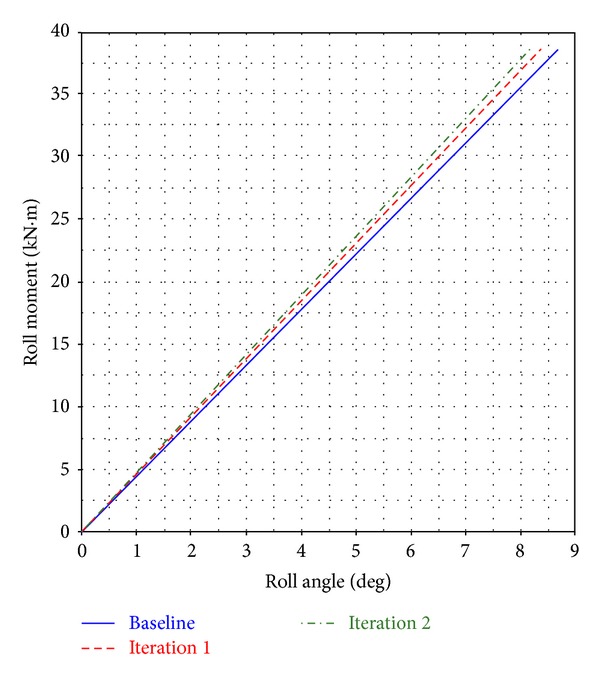
Graph of roll moment versus roll angle.

For the suspension roll study, a 1.5 g gravitational force is applied to the left side, and the load on the right side is decreased to 0.5 g of the gravitational force. In this case, the same antiroll bar, axle, and linkages are implemented to ensure consistency in the simulation. To determine suspension roll stiffness, the roll angle of the suspension is measured, as shown in Figure 8. The roll angle *θ* is measured based on the rotation of the solid axle in the *y*-axis connecting the left and the right parabolic leaf springs. The roll moment versus the roll angle curve for Baseline, Iteration 1, and Iteration 2 is plotted in Figure 9. The curve depicts an almost linear relation. The roll stiffness indicated by the gradient of the roll moment versus the roll angle curve and generated by the parabolic leaf spring in the Baseline is 4.46 kN·m/degree. The roll stiffness in Iteration 1 is 4.60 kN·m/degree, whereas that in Iteration 2 is 4.73 kN·m/degree. On the basis of the roll stiffness, the parabolic leaf spring in Iteration 2 contributes most to the roll stiffness of the suspension system, followed by Iteration 1 and then the Baseline. The suspension roll stiffness is closely associated with the vehicle body roll. Under the influence of the lateral inertia force, the vehicle body produces a roll angle Φ about the roll axis, approximately determined by

(11) $$\begin{array}{c} \Phi =\frac{M_{s}h_{\text{roll}}a_{y}}{k_{\Phi }}, \end{array}$$

where *M*_*s*_ is the vehicle sprung mass, *h*_roll_ is the height of the center of gravity of the vehicle body above the roll axis, and *k*_Φ_ is the total roll stiffness of suspension and tires [27]. According to (11), the vehicle body roll is inversely proportional to the suspension roll stiffness, with the suspension roll stiffness defined as follows:

(12) $$\begin{array}{c} k_{\Phi }=k_{f}+k_{d}+k_{r}, \end{array}$$

where the *k*_*f*_ is the front suspension roll stiffness, *k*_*d*_ is the device roll stiffness such as antiroll bar, and *k*_*r*_ is the rear suspension roll stiffness. The suspension front, rear roll stiffness, and the contribution of the antiroll bar constitute the amount of the vehicle body roll; thus, an increase in any of them reduces the vehicle body roll. The vehicle body roll reduces the stabilizing moment because of insufficient roll stiffness, leading to vehicle instability. Therefore, the parabolic leaf springs in Iteration 2 exhibit the highest suspension roll stiffness compared with those in Iteration 1 and the Baseline, thereby providing the vehicle the highest roll stability.

**Figure 10:**
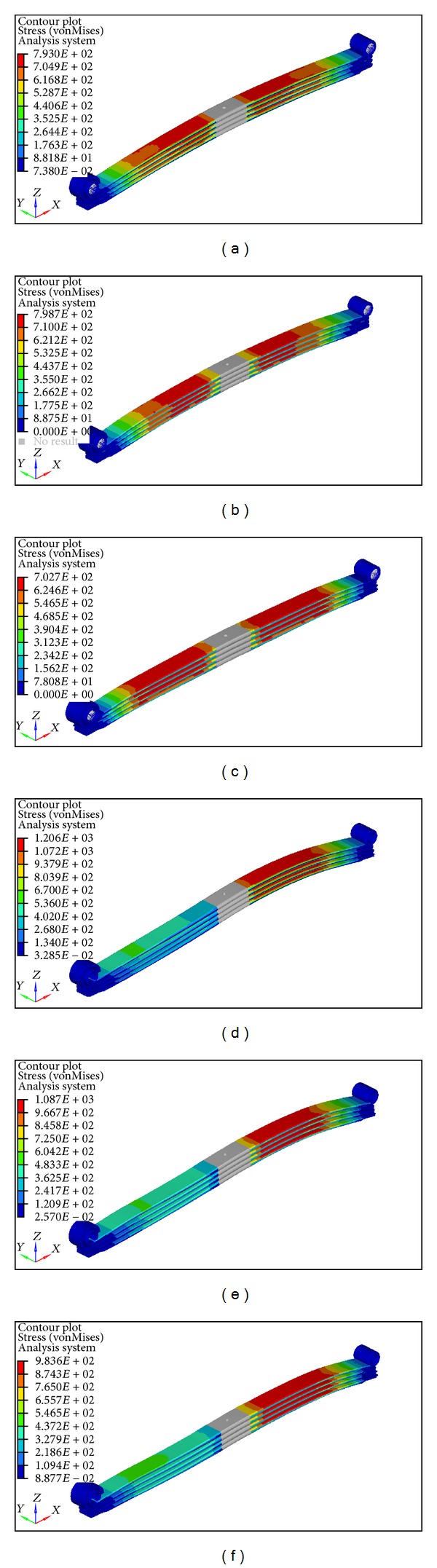
von Mises stress contour of parabolic leaf springs: (a) Baseline model vertical push, (b) Iteration 1 vertical push, (c) Iteration 2 vertical push, (d) Baseline model wind-up, (e) Iteration 1 wind-up, and (f) Iteration 2 wind-up.

**Figure 11:**
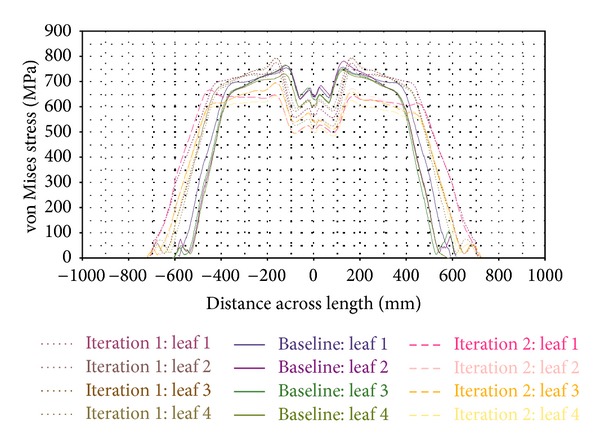
von Mises stress across length plot of vertical push.

**Figure 12:**
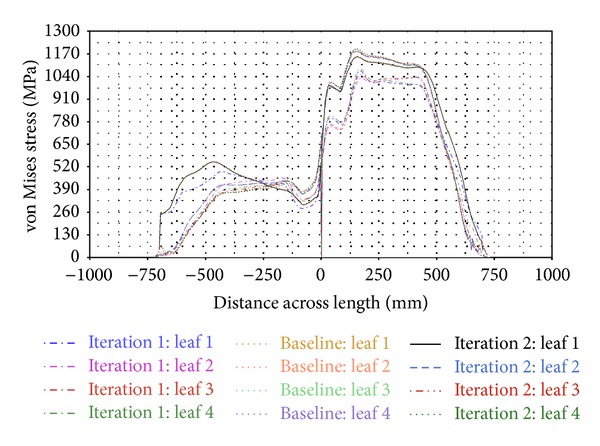
von Mises stress across length of wind-up loading.

External loads applied to a component, particularly springs that undergo repeated cyclic loading, produce stress. In real-life settings, stresses would not be uniaxial, biaxial, and/or even multiaxial for most cases. Alternatively, an equivalent stress can be calculated from multiaxial stresses. The von Mises stress is a widely known equivalent stress, which is implemented for stress analysis of the leaf spring in this study. The stress levels of machine components are often monitored and controlled within the limit of the material that can sustain stress to prevent component failure. The von Mises stress contours of the Baseline, Iteration 1, and Iteration 2 of parabolic leaf springs under vertical and wind-up load cases are illustrated in Figure 10. To improve the visualization of stress analysis, a comparison of von Mises stress across the length of the leaf spring for vertical push is plotted and shown in Figure 11. The von Mises stress of parabolic leaf springs under wind-up loading is plotted in Figure 12. The stress level of each leaf in the Baseline, Iteration 1, and Iteration 2 can be clearly visualized and compared. As shown in Figures 10 and 11, the overall von Mises stress level of the parabolic leaf springs ranges from 500 MPa to 800 MPa at the region 200 mm to 400 mm away from the center of the spring. The highest von Mises stress level of the first leaf until the fourth leaf of the parabolic leaf spring in Iteration 1 ranges from 700 MPa to 800 MPa. The stress level of the Baseline ranged from 650 MPa to 750 MPa in the high-stress region. Iteration 2 exhibits the lowest von Mises stress from about 600 MPa to 700 MPa, under the same load, followed by the Baseline; however, the highest stress is shown by Iteration 1. For wind-up analysis, the von Mises stress for the Baseline ranged from 1000 MPa to 1200 MPa for all leaves of the parabolic leaf spring. The stress is evenly distributed during the wind-up load case for Baseline. Under the same load, the von Mises stress for Iteration 2 is also distributed from 1000 MPa to 1200 MPa. The stress level for Iteration 1 ranged from 1040 MPa to 1080 MPa. The variation in stress level is typically small when the Baseline is compared with Iteration 1. In the wind-up cases, the parabolic leaf spring of Iteration 1 has a narrower stress range and amplitude compared with those of the Baseline and Iteration 2. The entire stress distribution can be affected by the design taper profile of the cantilever of the parabolic spring itself. However, the entire simulation model for Baseline, Iteration 1, and Iteration 2 remains within acceptable limits with an even stress distribution. Iteration 2 contributes the highest value of wind-up stiffness.

**Figure 13:**
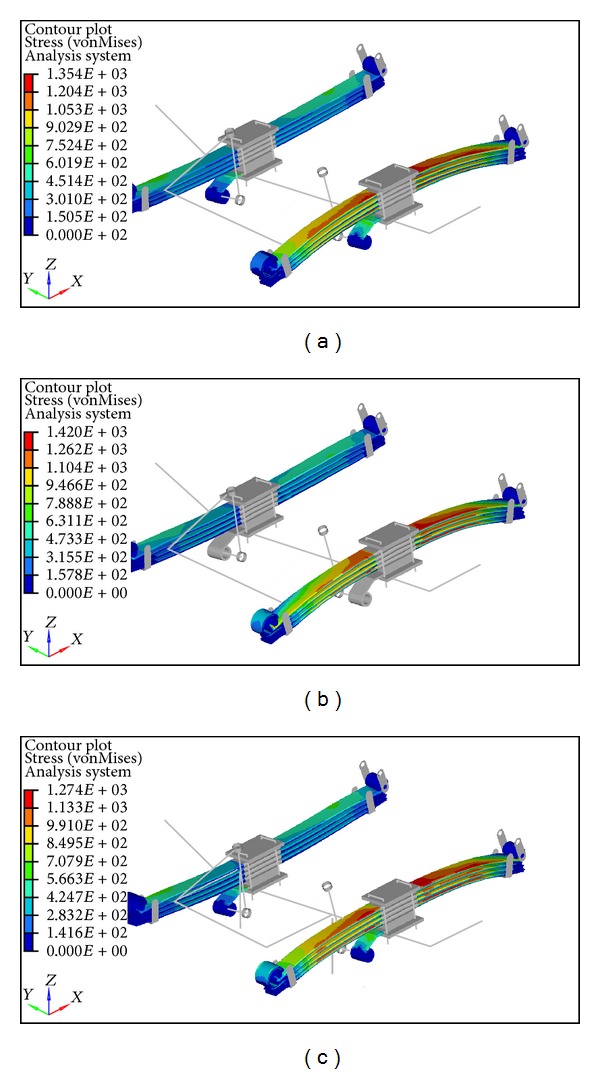
von Mises stress contour of parabolic leaf springs under roll load case: (a) Baseline model, (b) Iteration 1, and (c) Iteration 2.

**Figure 14:**
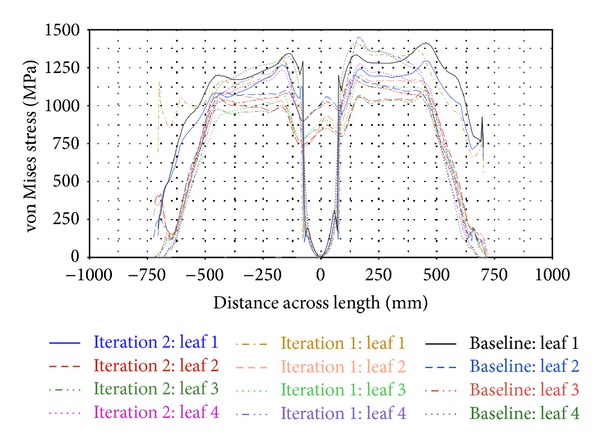
von Mises stress across length plot of roll load case.

Figure 13 shows the von Mises stress contours of the parabolic leaf springs when the roll load case is applied. The highest stress level is observed at the outer edge of the parabolic leaf spring during suspension under roll loading. The stress levels of all parabolic leaf spring variants are then plotted into a graph in Figure 14, which reveals that the main leaf and leaf 4 of Iteration 1 obtain the maximum range of the von Mises stress amplitude ranging from 1200 MPa to 1450 MPa. The remaining leaves ranged from 1000 MPa to 1200 MPa. By comparing the von Mises stress of the simulation, the level of stress of this roll loading approaches the yield strength of the material, which is 1502 MPa [28]. Iteration 1 is found to possess a very low safety factor under this condition. For the Baseline, the stress values of leaves 1 and 4 ranged from 1200 MPa to 1400 MPa, whereas those of leaves 2 and 3 ranged from 1000 MPa to 1200 MPa. The stress ranges of the Baseline and Iteration 1 are almost the same. The design of the Baseline model has a low safety factor. Finally, the stress contour of leaves 1 and 4 of Iteration 2 is also plotted, with the stress amplitude ranging from 1100 MPa to 1300 MPa. Meanwhile, the stress levels of leaves 2 and 3 range from about 900 MPa to 1100 MPa in the high-stress region. A 100 MPa stress reduction is observed when Iteration 1 and the Baseline are compared. The safety factor of the parabolic leaf spring of Iteration 2 is higher compared with those of Iteration 1 and the Baseline in this case. The parabolic leaf spring in Iteration 2 has a lower probability of failure under this load case compared with those of Iteration 1 and the Baseline. 

In a vertical load case, Iteration 2 exhibits higher vertical stiffness compared with both Iteration 1 and the Baseline. In addition, Iteration 2 possesses a higher resistance to longitudinal loading compared with Iteration 1 and the Baseline during wind-up loading. The roll stiffness of Iteration 2 is also slightly greater than those of Iteration 1 and the Baseline. The stress level of Iteration 2 is lower than those of Iteration 1 and the Baseline even in the case of vertical and roll loads, as listed in Table 2. The parabolic leaf spring in Iteration 2 must be able to successfully sustain the load for a longer period. However, a low-stiffness spring is favorable for the ride dynamics of any ground vehicle, which is often a compromise with vehicle handling. The latter usually prefers a high-stiffness spring. To identify the most suitable parabolic leaf spring design, many other factors should be considered, depending on the application and user perception of the vehicle. Nevertheless, the parabolic leaf spring in Iteration 2 with the highest vertical stiffness is shown to be the most suitable based on the load case simulation results.

## 5. Conclusions

An explicit dynamic nonlinear geometric scheme was adopted to simulate the vertical push, wind-up, and roll load cases of the parabolic leaf spring of a bus. An FE-based procedure dealing with the evaluation and assessment of the parabolic leaf spring of the bus was presented. Modeling details for an accurate calculation of the spring are discussed. New parabolic leaf spring designs are included in the analysis to obtain an improved bus load-carrying capability, braking resistance, and roll resistance, which were determined through the analysis of vertical stiffness, wind-up stiffness, and roll stiffness. In addition to the vertical, wind-up, and roll stiffness provided by the parabolic leaf springs, the stress level of the spring component itself is plotted and monitored to ensure falling within the controlled limit. Hence, no failures are expected when the new parabolic leaf spring designs are implemented in the vehicle. In this analysis, the designed parabolic leaf spring with higher vertical stiffness leads to higher wind-up and roll stiffness. The new parabolic leaf spring design with the highest vertical stiffness should possess higher load-carrying capability, braking instability resistance, and roll stability compared with the others. The stress level observed for the new leaf spring designs under these circumstances is lower compared with the original design. The chances of failure are reduced, and vehicle safety is enhanced under a braking or pothole strike condition. Vehicle safety is increased because of the increase in suspension reliability.

## Figures and Tables

**Table 1:** Materials and properties of leaf springs and silencers.

	Leaf springs	Silencers
Modulus of elasticity, *E*, GPa	210	4
Density, kg/m^3^	7850	900
Poisson ration	0.3	0.3

**Table 2:** Summary of vertical, wind-up, and roll stiffness and stress for Baseline, Iteration 1, and 2.

	Vertical stiffness (N/mm)	Wind-up stiffness (kN·m/degree)	Roll stiffness (kN·m/degree)	Maximum vertical stress (MPa)	Maximum wind-up stress (MPa)	Maximum roll stress (MPa)
Baseline	295	1.82	4.46	800	1198	1450
Iteration 1	281	2.04	4.60	750	1083	1400
Iteration 2	338	2.42	4.73	700	1198	1300
